# Signet-Ring Cell Colorectal Cancer and Signet-Ring Cell Component Colorectal Cancer: Do They Differ in Clinical Behavior?

**DOI:** 10.3390/jcm15072736

**Published:** 2026-04-04

**Authors:** Cebrail Akyuz, Oguzhan Sunamak, Aytac Selman, Mehmet O. Gul, Umut Kina Kilicaslan, Kadir Corbaci, Feza Ekiz, Turgut Donmez, Evren Besler

**Affiliations:** 1 Department of General Surgery, Haydarpasa Numune Training and Research Hospital, University of Health Sciences, Istanbul 34668, Turkey; o.sunamak@yahoo.com.tr (O.S.); aytacselman@gmail.com (A.S.); evrenbesler@hotmail.com (E.B.); 2 Department of General Surgery, Gaziantep City Hospital, University of Health Sciences, Gaziantep 27500, Turkey; mehmetonurgul@hotmail.com; 3 Department of General Surgery, Martin Luther Hospital, 14193 Berlin, Germany; 4 Department of General Surgery, Bilecik Seyh Edebali University, Bilecik 11100, Turkey; kadir.corbaci@bilecik.edu.tr; 5 Department of General Surgery, Istanbul Medical Faculty, University of Istanbul, Istanbul 34093, Turkey; fezaekiz@gmail.com; 6 Department of General Surgery, Bakirkoy Sadi Konuk Training and Research Hospital, University of Health Sciences, Istanbul 34149, Turkey; surgeont73@hotmail.com

**Keywords:** colon, colorectal carcinoma, prognosis, rectum, signet-ring cell

## Abstract

**Background**: Signet-ring cell colorectal cancer (SRCC) and colorectal cancer with a signet-ring cell component (SRC) are rare histological subtypes associated with poor prognosis. Although these entities are classified separately based on pathological criteria, it remains unclear whether they differ in clinical behavior and survival outcomes. This study aimed to compare the clinicopathological characteristics and survival of patients with SRCC and SRC. **Methods**: Patients who underwent surgery for colorectal cancer (CRC) between January 2012 and December 2019 and were diagnosed with SRCC or SRC on histopathological examination were retrospectively included. Demographic, clinical, and survival data were analyzed and compared between the two groups. **Results**: A total of 32 patients (22 males, 10 females) with SRCC or SRC were included, representing 2.6% (32/1253) of all CRC cases operated at our center. Seven patients (five with SRCC and two with SRC) had synchronous distant metastases, including liver-only metastases (9%), diffuse intra-abdominal seeding (9%), and ovarian metastasis (3%). The mean disease-free survival was 19.2 ± 3.3 months overall, and 17.18 ± 3.61 months for SRCC and 19.3 ± 9 months for SRC. The mean overall survival was 70.9 ± 10.18 months overall, and 65.95 ± 13.77 months for SRCC and 79.20 ± 15.03 months for SRC. No statistically significant difference in overall survival was observed between the groups (*p* = 0.534). **Conclusions**: SRCC and SRC demonstrate broadly similar clinicopathological features, with younger age at diagnosis observed in SRCC patients. However, given the limited sample size, the absence of statistically significant differences in survival outcomes should be interpreted with caution and does not imply equivalence. Distant metastasis remains the most important prognostic factor in both groups.

## 1. Introduction

Colorectal cancers (CRCs) have been an important public health problem in both incidence and mortality [1]. However, in spite of not being a homogenous disease, CRCs show heterogeneity at the histopathological and molecular levels [2]. This heterogeneity directly affects clinical progress, response to treatment, and prognosis. In this aspect, being a rare but clinically aggressive subtype, signet-ring cell cancer (SRCC) has gained increasing attention in recent years [3].

SRCC is defined by its unique morphology, which shows displacement of the nucleus to the periphery because of the accumulation of mucin in the cytoplasm. According to World Health Organization criteria, this phenotype must be present in at least 50% of tumor cells [4]. Although colorectal SRCCs consist of less than 1% of all CRCs, they demonstrate prominently different clinical and biological properties compared to the classic CRCs [3,5]. On the other hand, the clinical significance of descriptive threshold values for the adenocarcinomas having less than 50% signet-ring cell component in tumor tissue (tumors with SRC (SRC)) is still controversial, and there are conflicting results on the prognostic importance of this group in the literature [1].

Contemporary data show that SRCCs tend to be diagnosed at a younger age, being presented at an advanced stage, and having a diffusely infiltrating growth pattern [5,6].

Also, it has been reported that peritoneal dissemination, lymph node invasion, and distant metastasis rates are higher, thus resulting in worse overall survival rates [6,7]. But, it is proposed that the prognosis may change depending on the ratio of signet cell component, molecular properties, and accompanying histopathological pattern of the tumor with SRC; thus, treating this group as a single clinical entity may be insufficient [1].

In the molecular aspect, the microsatellite instability of SRCC was shown to be related to CpG island methylation phenotype and loss of e-cadherin which plays a role in cell adhesion [2,8,9]. However, the molecular pathogenesis of these tumors has not been fully explained, and it is thought that alternative mechanisms which are partially independent from classical carcinogenesis pathways [4,10].

It was reported that the molecular profile of SRC tumors, however, is more heterogeneous and might have different biological behavior on the basis of signet-ring cell ratio [1,3]. This situation brings the question to mind whether the current classification systems are sufficiently discriminative in the clinical decision-making process.

SRCC and colorectal cancers with SRCs result in an important therapeutic difficulty in clinical management. It was reported that these types of tumors might have a restricted response to the standard chemotherapy regimens, lower surgical resection rates due to diagnosis at an advanced stage, and worse overall survival rates [7,11]. But the optimal treatment strategies specific to this group of patients have not been clarified yet, and current guidelines do not specifically deal with these rare subtypes enough [12,13,14].

In this aspect, a more detailed understanding of the clinicopathological and prognostic properties of SRCC and SRC is needed. This study aims to analyze and compare the clinical and pathological features of SRCC and SRC, and to find out the potential effect of the signet-ring cell ratio on prognosis.

## 2. Materials and Methods

This retrospective study was approved by the institutional ethics committee (approval number: E-62977267-903.99; date: 30 March 2021). Patients who underwent surgery for colorectal cancer between January 2012 and December 2019 and were diagnosed with signet-ring cell colorectal cancer (SRCC) or colorectal cancer with a signet-ring cell component (SRC) on histopathological examination were included in the study.

Patients were identified from the institutional colorectal cancer database, and all consecutive eligible cases were included to minimize selection bias. Histopathological classification into SRCC and SRC was performed according to established criteria based on the proportion of signet-ring cells (≥50% for SRCC and <50% for SRC). All pathological evaluations were carried out by experienced pathologists as part of routine clinical practice. Cases with incomplete histopathological data or non-eligible tumor subtypes were excluded. No missing histological classification data were present among the included patients.

**Figure 1:**
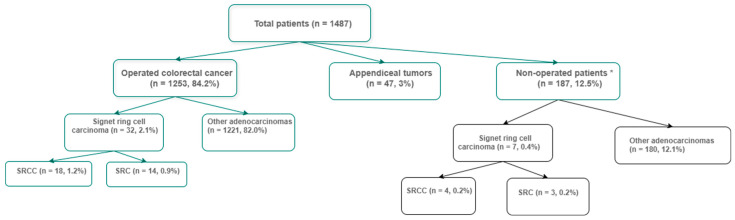
Flow diagram. (* medically inoperable, surgically irrsecetable disease).

Inoperative cases and patients with appendiceal SRCC were excluded. A flowchart of patient selection is presented in Figure 1. Tumor staging and grading were performed according to the TNM classification and the American Joint Committee on Cancer (AJCC) 2017 guidelines [15].

The following variables were recorded: age, sex, tumor localization, tumor markers, presenting symptoms, surgical procedure, tumor histology, administration of chemotherapy and radiotherapy, disease-free survival (DFS), and overall survival (OS). Treatment decisions, including adjuvant chemotherapy and neoadjuvant chemoradiotherapy (NCRT), were made based on standard institutional protocols and multidisciplinary team evaluation. Chemotherapy regimens primarily consisted of fluoropyrimidine-based combinations such as FOLFOX or CAPOX, depending on tumor stage, patient characteristics, and clinician judgment. Due to the retrospective design, treatment allocation was not fully standardized.

Tumors located in the cecum, ascending colon, and proximal two-thirds of the transverse colon were classified as right-sided tumors, whereas tumors located in the descending colon, sigmoid colon, and rectum were classified as left-sided tumors. Additional histopathological features, including TNM stage, perineural invasion, lymphovascular invasion, mucinous component, and tumor deposits, were also analyzed. MSI status was assessed based on routine clinical records.

### Statistical Analysis

Statistical analysis was performed using IBM SPSS Statistics for Windows, version 25.0 (IBM Corp., Armonk, NY, USA). Sample size was calculated using RStudio, version 2024.12.1 + 563 (Posit Software, PBC, Boston, MA, USA) and Quarto, version 1.5.57 (Posit Software, PBC, Boston, MA, USA) in the R environment, based on the log-rank test using the Lachin–Foulkes method implemented in the gsDesign package (nSurv() function). A conservative hazard ratio (HR) of 3 was selected based on previously published data, with a two-sided significance level (α) of 0.05 and 80% statistical power [16]. Accordingly, a minimum of 24 patients was required; after accounting for a potential 20% dropout rate, the target sample size was set at 30 patients.

Categorical variables were expressed as numbers and percentages, while continuous variables were presented as mean ± standard deviation or median (minimum–maximum), as appropriate. The Kolmogorov–Smirnov test was used to assess normality.

Overall survival (OS) was defined as the time from diagnosis to death from any cause. Disease-free survival (DFS) was defined as the time from surgery to recurrence or death. The median follow-up time was 33 months (range: 0–108 months). Patients without events were considered right-censored. Survival analyses were performed using the Kaplan–Meier method and compared using the log-rank test. Median survival times and corresponding intervals were calculated accordingly.

Logistic regression analysis was used to evaluate factors associated with the presence of SRCC. A *p*-value < 0.05 was considered statistically significant. Molecular variables such as MSI and RAS were not included in regression models due to the limited number of patients with available data, in order to avoid overfitting and unstable estimates.

## 3. Results

Demographic and clinical characteristics of the patients are summarized in Table 1. A total of 32 patients (22 males and 10 females) with SRCC or SRC were included, representing 2.55% (32/1253) of all colorectal cancer cases operated at our center during the study period. Among these, 18 patients were classified as SRCC and 14 as SRC.

The mean age of the cohort was 57.8 ± 13.2 years. The median age was lower in the SRCC group compared to the SRC group [54.5 (range: 39–80) vs. 65 (range: 32–76) years, respectively].

Synchronous distant metastases were present in seven patients, including five patients (71.4%) in the SRCC group and two patients (28.6%) in the SRC group. Among these, three patients had liver-only metastases (9%), three had diffuse intra-abdominal dissemination (9%), and one patient had ovarian metastasis (3%).

Age, sex, tumor localization, stage, grade, perineural and lymphovascular invasion, presence of mucin, and tumor deposits are presented in Table 1. Among these variables, only age showed a statistically significant difference between the groups, with patients in the SRCC group being younger.

None of these clinicopathological parameters were significantly associated with survival in univariate or multivariate analyses, except for the presence of distant metastasis, which was identified as a significant prognostic factor (Table 2).

RAS mutations were detected in three patients, including one with an NRAS mutation and two with KRAS mutations.

**Figure 2:**
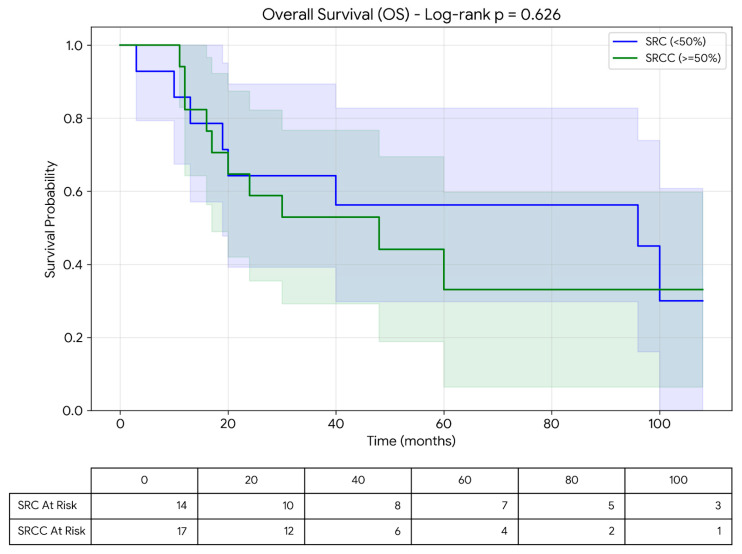
Kaplan–Meier analysis of overall survival between SRCC and SRC, including number at risk table.

Microsatellite instability (MSI) status was available for 27 patients. High MSI (MSI-high) was identified in 24 patients. Among the SRC group, MSI was evaluated in 16 patients and was found to be high in 13 (81.3%). The median survival time of SRC patients with MSI-high status was 24 months (range: 0–108 months). In the SRCC group, MSI status was assessed in 11 patients, all of whom were found to have MSI-high tumors. The median survival time of SRCC patients with MSI-high status was 72 months (range: 10–108 months). The observed MSI-high rate in our cohort was notably higher than that typically reported in unselected colorectal cancer populations. This finding may be related to the specific histological subtypes included in this study as well as the limited sample size. The mean disease-free survival (DFS) for the entire cohort was 19.2 ± 3.3 months, and 17.18 ± 3.61 months for SRCC and 19.3 ± 9 months for SRC, respectively. No statistically significant difference was observed between the groups (*p* = 0.834). The mean overall survival (OS) was 70.9 ± 10.18 months for all patients, and 65.95 ± 13.77 months for SRCC and 79.20 ± 15.03 months for SRC, respectively. No statistically significant difference in overall survival was observed between the groups (*p* = 0.534) (Figure 2).

**Figure 3:**
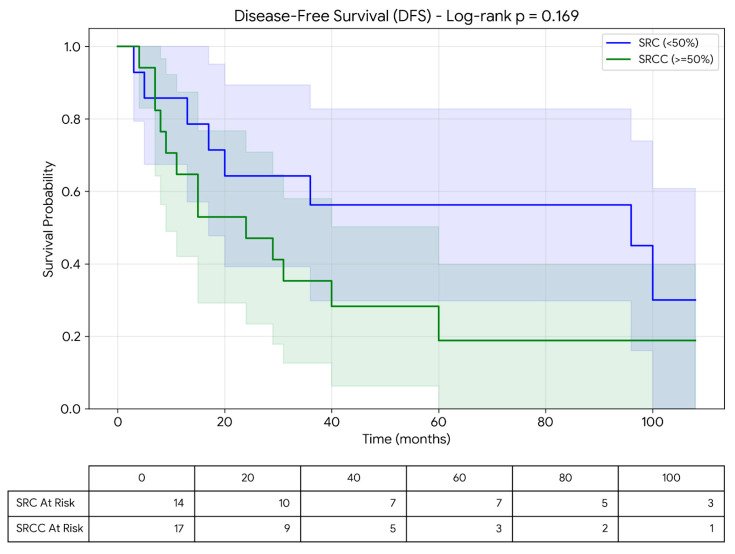
Kaplan–Meier analysis of disease-free survival between SRCC and SRC, including number at risk table.

**Figure 4:**
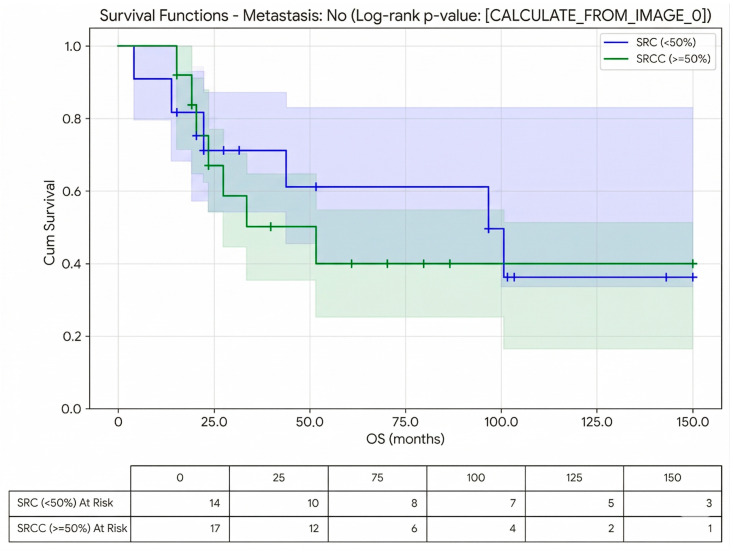
Kaplan–Meier analysis of overall survival between SRCC and SRC in non-metastatic patients, including number at risk table.

Univariate analysis of clinicopathological parameters demonstrated that the presence of distant metastasis was significantly associated with survival (*p* = 0.007, HR = 4.6, 95% CI: 1.5–14) (Figure 3 and Figure 4; Table 2).

Carcinoembryonic antigen (CEA) levels were elevated in 20 patients (62.5%), including nine patients (45%) in the SRCC group and 11 patients (55%) in the SRC group (*p* = 0.716). The median survival time of SRC patients with elevated CEA levels was 16 months (range: 0–60 months), whereas it was 24 months (range: 3–108 months) in SRCC patients.

Tumor location was right-sided in 10 patients (31.3%) and left-sided or rectal in 22 patients (68.8%).

Ten patients underwent emergency surgery due to mechanical intestinal obstruction, including six patients (33.3%) in the SRCC group and four patients (28.6%) in the SRC group (*p* = 0.541). Among the remaining patients, eight (25%) presented with anemia, 10 (31.3%) with abdominal pain, and four (12.5%) with dyspeptic symptoms.

Although no patient had macroscopic perforation, histopathological examination revealed microscopic perforation in three patients (16.7%) in the SRCC group and one patient (7.1%) in the SRC group.

Adjuvant chemotherapy was administered to 29 patients postoperatively, while three patients did not receive it. In the SRCC group, one patient did not receive chemotherapy due to advanced age and comorbidities. In the SRC group, one patient had stage I disease and another could not tolerate chemotherapy. The distribution of chemotherapy administration was comparable between the SRCC and SRC groups. Due to the limited sample size, a detailed comparison of specific chemotherapy regimens between groups was not feasible. These findings should be interpreted cautiously given the limited sample size.

All six patients with rectal cancer received neoadjuvant chemoradiotherapy (NCRT), including three patients with SRCC and three with SRC. The median follow-up period for these patients was 44.4 months (range: 12–96 months). During follow-up, one patient developed recurrence at month 15 and died five months after receiving adjuvant chemotherapy.

Survival analysis according to SRCC and SRC subtypes is presented in Table 3.

## 4. Discussion

SRCC and SRC are rare histological subtypes of colorectal cancer. While SRCC is generally associated with a poorer prognosis, SRC is often considered within the spectrum of conventional colorectal adenocarcinomas [2,3]. In the present study, no statistically significant difference was observed between SRCC and SRC in terms of survival outcomes, except for the younger age at diagnosis in the SRCC group.

Previous studies have reported that SRCC tends to occur more frequently in younger patients, particularly those under 40 years of age, although some series have included older populations [16,17,18]. In our study, SRCC patients were significantly younger than SRC patients, which is consistent with the existing literature.

Several studies have demonstrated that SRCC is associated with more advanced disease at diagnosis, including higher T stage, increased lymphovascular and perineural invasion, and poorer prognosis compared to conventional colorectal cancer [7,19]. In our cohort, most patients in both SRCC and SRC groups were diagnosed at advanced stages (T3–T4), which may partially explain the similar survival outcomes observed between the groups.

Recent studies comparing SRCC and SRC directly have reported no significant differences in clinicopathological features or survival outcomes [14,15]. Our findings are consistent with these reports, suggesting that the proportion of signet-ring cells alone may not be sufficient to distinguish clinically meaningful differences between these subtypes.

The distribution of tumor location in SRCC remains controversial. While some studies report a predominance in the right colon, others have found higher rates in the left colon or rectosigmoid region [18,20,21,22,23]. In our study, both SRCC and SRC were more frequently located in the left colon, with no significant difference between the groups.

In our study, no statistically significant difference in survival was observed between groups stratified by signet-ring cell (SRC) proportion (≥50% vs. <50%). However, this finding should be interpreted with caution. One of the key strengths of this study is the stratification of tumors based on SRC proportion. This distinction is clinically relevant, as SRC morphology is widely recognized as an aggressive histopathological phenotype associated with poor prognosis in colorectal cancer [6,20,24]. Nevertheless, it remains unclear whether the proportion of SRCs within a tumor has a direct impact on prognosis. Our findings suggest that SRC proportion alone may not be a primary determinant of clinical outcomes. In this context, the lack of a statistically significant difference observed in our study contributes to the existing literature by indicating that a higher SRC content does not necessarily translate into worse prognosis [21]. These results further support the concept that SRC morphology represents a distinct pathological phenotype characterized by inherently aggressive biological behavior. However, this aggressive behavior does not appear to be solely dependent on the proportion of SRCs. Rather, underlying molecular characteristics of the tumor may play a more decisive role in determining prognosis. Factors such as microsatellite instability, epigenetic alterations, and disruptions in cell adhesion mechanisms may act as key drivers of tumor biology, independent of morphological features [25,26,27].

In our study, a notably high frequency of microsatellite instability (MSI) was observed in patients with signet-ring cell colorectal cancer (SRCC/SRC). MSI-high status was identified in the majority of cases, supporting previous reports that suggest an association between signet-ring cell morphology and the MSI-high phenotype [4,28]. This high prevalence of MSI indicates that defects in the DNA mismatch repair system, leading to genomic instability and increased tumor heterogeneity, may play a central role in the pathogenesis of SRCC. In addition to MSI, previous studies have reported that BRAF mutations, CpG island methylator phenotype (CIMP), and alterations in chromatin remodeling pathways, including the SWI/SNF complex and INI-1, are frequently observed in SRCC. Collectively, these molecular abnormalities are thought to underlie both the aggressive clinical course and the distinctive histopathological features of this tumor subtype [1,28,29]. The relationship between signet-ring cell morphology and the underlying molecular genetic landscape is complex. While prominent intracellular mucin accumulation and peripheral nuclear displacement define the phenotype at the cellular level, these features do not fully capture the diversity of molecular events driving tumor progression. Our findings emphasize that morphological characteristics alone may be insufficient to predict clinical outcomes and highlight the importance of a comprehensive evaluation that incorporates molecular features. Furthermore, the molecular heterogeneity within SRCC may partly explain the variability in chemotherapy response observed in clinical practice. MSI-high tumors are generally considered less responsive to conventional fluoropyrimidine-based chemotherapy, yet they may exhibit a higher likelihood of response to immunotherapy [30]. Although epigenetic alterations and disruptions in chromatin remodeling pathways have also been proposed to influence drug sensitivity and resistance, the specific mechanisms underlying these effects in SRCC remain insufficiently elucidated [1,28,29]. By addressing this molecular complexity, our study provides additional insight into the biological behavior of SRCC. Future studies integrating histopathological classification with molecular profiling will be essential for a more comprehensive understanding of SRCC pathobiology and for optimizing patient-specific therapeutic strategies.

Previous studies have shown that MSI-high status is associated with enhanced immune response and may serve as a predictive biomarker for treatment with immune checkpoint inhibitors [31]. In this context, routine MSI testing in patients with SRCC may represent an important step toward identifying candidates for personalized immunotherapy. Moreover, early detection of MSI-high status is particularly relevant in advanced-stage patients with limited surgical options, as it may facilitate timely initiation of appropriate systemic therapies. Accordingly, the broader implementation of early molecular diagnostics and MSI testing may contribute to the development of more effective treatment strategies and ultimately improve the prognosis of patients with SRCC [32]. The strong association between SRCC and MSI-high observed in our study further underscores its clinical relevance, particularly in terms of integrating immunotherapy options and molecular diagnostics into routine clinical practice.

The prognostic role of mucinous components remains unclear. While some studies suggest that mucin-poor SRCC may have a worse prognosis compared to mucin-rich tumors [33,34], we did not observe a significant difference in mucin content between the groups, nor did mucin presence have a significant impact on survival.

Similarly, lymphovascular and perineural invasion have been reported to be more frequent in SRCC and associated with poorer outcomes [35]. However, in our study, no significant differences were observed between SRCC and SRC regarding these parameters, and neither had a significant impact on survival.

The current clinical guidelines do not suggest a specified treatment for SRCC [36,37]. However, it was reported that colorectal SRCC and SRC have a lower response to standard chemotherapy regimens, less curative resection ratio, and worse prognosis, thus, necessitating newer treatment approaches [38]. It was reported that adjuvant chemotherapy provides a longer survival in SRCC, especially for stage III colon tumors, though SRCC itself is an independent poor prognostic factor [7]. In our study, most patients received adjuvant chemotherapy, although treatment allocation was not fully standardized due to the retrospective design.

The positive effect of curative surgical resection of metastases on survival has been well-known [39]. However, this option is limited to liver and lung metastases, and there is no data on the effect of histology on the postoperative outcome. Also, SRCC patients more frequently develop peritoneal and ovarian metastases that are less relevant for curative surgery. Van Oudheusden et al. performed cytoreductive surgery and hyperthermic intraperitoneal chemotherapy (HIPEC) in 16 cases of colorectal SRCC with peritoneal carcinomatosis. They stated that macroscopic complete resection was achieved in 97.2% of the cases. Relapses occurred in 68.8% of SRCC, and the median survival was 14.1 months, while the median survival in the adenocarcinoma group was 35.1 months [40]. In our study, there was no significant difference between the distant metastasis rates of both groups. This may explain the similarity in the survival of both groups.

Neoadjuvant chemoradiotherapy (NCRT) remains the standard approach for rectal cancer. Rectal SRCC and SRC have been treated in the light of rectal cancer guidelines [41]. In our study, a small number of patients received NCRT, and due to this limited sample size, a formal analysis of its impact on survival was not feasible.

This study has several limitations. First, its retrospective design and relatively small sample size limit the generalizability of the findings. Second, treatment heterogeneity may have influenced survival outcomes. Third, the limited availability of molecular data restricted more comprehensive analyses. RAS mutation data were available only for a limited number of patients, precluding meaningful statistical analysis or inclusion in multivariable models. Although the sample size met the minimum requirement based on a priori calculations, the study may still be underpowered to detect smaller or moderate differences between groups. Therefore, the absence of statistically significant differences between SRCC and SRC should not be interpreted as evidence of equivalence. These findings should be considered hypothesis-generating and require validation in larger, multicenter cohorts.

## 5. Conclusions

In this retrospective cohort, SRCC and SRC demonstrated broadly comparable clinicopathological features, with younger age observed in the SRCC group. However, despite meeting the calculated sample size, the study may still be underpowered to detect moderate differences. Therefore, the absence of statistically significant differences in survival outcomes should be interpreted with caution and does not imply equivalence between the two groups. Larger, multicenter studies are needed to better define the prognostic significance of signet-ring cell proportion in colorectal cancer.

## Figures and Tables

**Table 1:** Demographic and clinicopathological characteristics of patients with SRCC and SRC.

	Percent of Signet-Ring Cells		
Parameters	<50 (SRC)	≥50 (SRCC)	*p*
**Age (year) ^a^** (mean 57.8 ± 13.2)	62.3 ± 11.2 65.0 (32–76)	54.7 ± 12.0 54.5 (39–80)	0.045 *
**Gender ^b^**			
Male	10 (71.4)	12 (66.7)	>0.999
Female	4 (28.6)	6 (33.3)	
**Stage**			
1	1 (7.1)	0 (0.0)	0.632
2	4 (28.6)	4 (22.2)	
3	7 (50.0)	10 (55.6)	
4	2 (14.3)	4 (22.2)	
**Location ^b^**			
(Cecum + Ascending colon + Hepatic Flexura)	5 (35.7)	5 (27.8)	0.859
(Descending colon + Rectosigmoid + Sigmoid + Splenic flexure + Rectum)	8 (57.1)	12 (66.7)	
Transverse colon	1 (7.1)	1 (5.6)	
**T classification ^b^**			
2	1 (7.1)	0 (0.0)	0.717
3	6 (42.9)	8 (44.4)	
4a	4 (28.6)	6 (33.3)	
4b	3 (21.4)	4 (22.2)	
**Lymph nodes metastasis ^b^**			
N0	6 (42.9)	5 (28.0)	0.566
N1	1 (7.1)	3 (17.0)	
N2	7 (50.0)	10 (56.0)	
**Distant metastasis ^b^**			
Yes	2 (14.3)	5 (27.8)	0.426
No	12 (85.7)	13 (72.2)	
**Perineural invasion ^b^**			
Yes	5 (35.7)	7 (38.9)	>0.999
No	9 (64.3)	11 (61.1)	
**Lymphovascular invasion ^b^**			
Yes	10 (71.4)	15 (83.3)	0.669
No	4 (28.6)	3 (16.7)	
**Tumor deposit ^b^**			
Yes	6 (50.0)	11 (61.1)	0.821
No	6 (50.0)	7 (38.9)	
**Mucinous component ^b^**			
Yes	6 (42.9)	6 (33.3)	0.854
No	8 (57.1)	12 (66.7)	
**Lymph nodes removed ^a^**	29.8 ± 15.22 6.5 (12–70)	34.9 ± 15.1 34 (12–60)	0.267
**Metastatic lymph nodes ^a^**	6.5 ± 8.6 3.5 (0–26)	9.6 ± 10 10.5 (0–34)	0.488

**Abbreviations:** SRCC, signet-ring cell carcinoma; SRC, signet-ring cell component. ^a^ Values are given as mean ± standard deviation and median (min-max), ^b^ Values are given as n (%), * Statistically significant (*p* < 0.05).

**Table 2:** Univariate and multivariate analyses of prognostic factors for survival in patients with SRCC and SRC (* means significant statistically).

Parameters		Univariate	
		HR (95% CI)	*p* Value
**Age**		1.0 (0.9–1.1)	0.309
**Gender**	Female	-	0.190
	Male	1.9 (0.7–4.9)	
**Mucinous component**	No	-	0.772
	Yes	0.9 (0.3–2.3)	
**Distant metastasis**	No	-	**0.007** *
	Yes	4.6 (1.5–14.0)	
**Perineural invasion**	No	-	0.569
	Yes	1.3 (0.5–3.4)	
**Lymphovascular invasion**	No	-	0.209
	Yes	2.2 (0.6–7.8)	
**Tumor deposit**	No	-	0.165
	Yes	2.0 (0.8–5.2)	

**Table 3:** Survival analysis of SRCC and SRC subtypes.

Outcome Measure	Group	N	Events	Mean Time (Months ± SD)	Median Time (Months)	Log-Rank (*p*)
Overall Survival (OS)	SRC (<50%)	14	8	55.79 ± 40.93	56.0	0.534
	SRCC (≥50%)	18	11	40.00 ± 29.36	30.0	
Disease-Free Survival (DFS)	SRC (<50%)	14	8	55.00 ± 41.64	54.0	0.834
	SRCC (≥50%)	18	14	32.47 ± 31.00	24.0	

## Data Availability

The data presented in this study are available on request from the corresponding author due to ethical and privacy restrictions.

## Abbreviations

The following abbreviations are used in this manuscript:

SRCC	Signet-ring cell colorectal cancer
CRC	Colorectal cancers
SRC	Signet-ring cell
MSI	Microsatellite instability
CEA	Carcinoembryonic antigen
NCRT	Neoadjuvant chemoradiotherapy
